# Medicaid expansions and differences in guideline‐adherent cervical cancer screening between American Indian and White women

**DOI:** 10.1002/cam4.5593

**Published:** 2023-01-11

**Authors:** Danielle R. Gartner, Jessica Y. Islam, Claire E. Margerison

**Affiliations:** ^1^ Department of Epidemiology and Biostatistics, College of Human Medicine Michigan State University East Lansing Michigan USA; ^2^ Cancer Epidemiology Program H. Lee Moffitt Cancer Center and Research Institute Tampa Florida USA; ^3^ Center for Immunization and Infection Research in Cancer H. Lee Moffitt Cancer Center and Research Institute Tampa Florida USA; ^4^ Department of Oncologic Sciences University of South Florida Tampa Florida USA

**Keywords:** American Indians or Alaska Natives, early detection of cancer, healthcare disparities, Medicaid, Papanicolaou test

## Abstract

**Background:**

Although preventable through screening, cervical cancer incidence and mortality are higher among American Indian and Alaska Native women (AIAN) than White women. The Patient Protection and Affordable Care Act's (ACA) Medicaid expansions may uniquely impact access and use of cervical cancer screening among AIAN women and ultimately alleviate this disparity.

**Methods:**

Using Medicaid eligible AIAN (*N* = 4681) and White (*N* = 57,661) women aged 18–64 years from the 2010–2020 Behavioral Risk Factor Surveillance System, we implemented difference‐in‐differences regression to estimate the association between the Medicaid expansions and guideline‐adherent cervical cancer screening and health care coverage.

**Results:**

The Medicaid expansions were not associated with guideline‐adherent cervical cancer screening (AIAN: −1 percentage point [ppt] [95% confidence interval, CI: −4, 2 ppts]; White: 3 ppts [95% CI: −0, 6 ppts]), but were associated with a 2 ppt increase (95% CI: 0, 4 ppt) in having had a pap test in the last 5 years among White women. The Medicaid expansions were also associated with increases in having a health plan (AIAN: 5 ppts [95% CI: 1, 9]; White: 11 ppts [95% CI: 7, 15]) and decreases in avoiding medical care due to costs (AIAN: −8 ppts [95% CI: −13, −2]; White: −6 ppts [95% CI: −9, −4]).

**Conclusions:**

While we observed improvements in health care coverage, we did not observe changes to guideline‐adherent cervical cancer screening following the ACA's Medicaid expansions. Given the disproportionate burden of cervical cancer among AIAN women, identifying ways to improve cervical cancer screening uptake and delivery should be prioritized to reduce preventable deaths.

## Graphical abstract

While preventable through screening, excess cervical cancer burdens the American Indian and Alaska Native (AIAN) population of the United States. Specifically, AIAN women are more likely to be diagnosed at advanced cancer stages than White women, suggesting AIAN experience barriers to timely pap tests or human papillomavirus (HPV) testing. Screening could be low because of insurance coverage and cost‐related barriers, which the Affordable Care Act's (ACA) Medicaid expansions potentially addressed. However, our study results suggest that increases in Medicaid coverage did not lead to more pap tests for AIAN women.

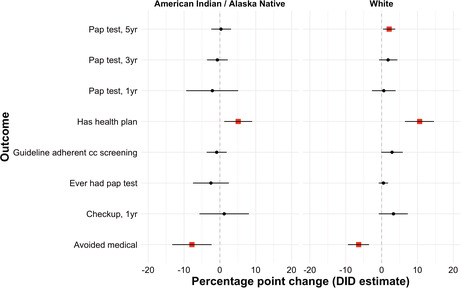

## 1 INTRODUCTION

The U.S. Patient Protection and Affordable Care Act's (ACA) Medicaid expansions, which began January 1st, 2014, provide the potential to impact American Indian and Alaska Native (AIAN) health.^1^, ^2^ Individually, AIAN who live at or below the 138% federal poverty level (FPL) in Medicaid expansion states can enroll and gain access to benefits and providers who accept Medicaid. At a system level, because many AIAN live at or below 138% FPL,^3^ increasing Medicaid coverage among AIAN can also contribute to increased reimbursement revenue for Indian Health Service (IHS) facilities, allowing additional services to be offered by this chronically underfunded institution.^2^, ^3^, ^4^ The IHS is a federal system of clinics and hospitals that are operated either federally or by tribes that provide health‐related services to members of federally recognized tribes. AIAN hold a unique political status and legal basis for healthcare services which is rooted in government‐to‐government relationships and treaties, also known as the Federal Trust Responsibility.^4^, ^5^ The Indian Health Service is the primary way that the United States (U.S.) upholds its Federal Trust Responsibility.

Despite administrative challenges to gaining Medicaid coverage,^6^ AIAN experienced an increase in public insurance and a decrease in uninsurance around the time of the ACA's Medicaid expansions.^7^, ^8^ However, limited evidence exists regarding if and how increases in insurance coverage among AIAN translate to changes in healthcare use,^9^ including cancer preventive services. Although highly preventable through screening and treatment,^10^ cervical cancer incidence and mortality are higher among AIAN than White women.^11^, ^12^ Because cervical cancer is caused by human papillomavirus (HPV), cervical cancer can be prevented through screening (e.g., Papanicolaou [pap] and HPV testing), and indeed, pap testing has contributed to declines in cervical cancer rates.^13^, ^14^ However, AIAN women are more likely to be diagnosed at advanced stages compared to White women,^15^ suggesting AIAN experience significant barriers to timely screening. Among AIAN served by IHS, screening is about 34%,^16^, ^17^ while it is 77%^18^ for self‐reported AIAN, both of which fall short of the Healthy People 2030 goal of 84.3%. Cervical cancer screening levels could be insufficient because of insurance coverage and several cost‐related barriers,^18^ which the Medicaid expansions potentially addressed.

Our primary research aim, therefore, was to estimate the impact of the ACA's Medicaid expansions on guideline‐adherent cervical cancer screening among AIAN and White women. We hypothesized that both AIAN and White women would experience an increase in guideline‐adherent cervical cancer screening, though the increase would be greater for AIAN because of the added mechanism of IHS reimbursement.

## 2 METHODS

### 2.1 Study sample

Data were from the 2010–2020 Behavioral Risk Factor Surveillance System (BRFSS),^19^ a nationally representative, yearly survey coordinated by the Centers for Disease Control and Prevention to monitor causes of morbidity among non‐institutionalized adults in the U.S. We included data from even years to accommodate the bi‐yearly breast and cervical cancer and HPV modules. The target population included people self‐identifying as female and non‐Hispanic (NH) AIAN only or NH White only (herein referred to as AIAN or White), aged 18–64 years, that resided in one of the 50 states in the U.S., or the District of Colombia, that also met the 138% FPL for Medicaid eligibility. To determine eligibility, we compared reported household size (adults and children) and annual household income to the appropriate, state‐, household size‐ and year‐specific 138% FPL values and excluded those likely not eligible for Medcaid.^20^ We then excluded people with hysterectomy. We performed a complete case analysis. The unweighted analytic sample included 4681 AIAN people and 57,661 White people (Figure S1). Due to variable missingness of outcome variables, the sample size used for each outcome model is provided in Table S1.

### 2.2 Measures

Exposed people were those residing in one of the 37 states that expanded Medicaid between January 1, 2014, and December 31, 2020 (Table S2). Unexposed people were those residing in the remaining 14 states that did not expand Medicaid between those dates. We used the date of the interview to categorize as pre‐ or post‐expansion and required people to have experienced at least 1 year of the expansion to be categorized as post expansion.

The primary outcome was receipt of guideline‐adherent cervical cancer screening, the criteria of which was recommended for update in 2012.^21^, ^22^ Therefore, for years 2010–2012, guideline‐adherent screening was indicated by having had a pap test in the last 3 years. For 2014 and later, guideline‐adherent screening was indicated as having had no pap test for those ages 18–24 and having had a pap test in the last 3 years for those aged 25–29 years. For those aged 30–65 years, guideline adherence was determined either by having had a pap test in the last 3 years or by having had a combination of a pap test and an HPV test within the last 5 years.

We included seven secondary outcomes. Four pap‐specific questions included having had a pap test in the last year, as well as having had a pap test within the last 3 years, within the last 5 years, or ever having had a pap test. We also included three health care use outcomes: having a health plan, having had a routine check‐up in the last year, and having avoided medical care in the last year due to cost.

In addition to using state and year fixed effects, covariates included individual‐level education level (less than high school diploma, high school diploma or equivalent, some college or technical school, college degree or more), yearly household income (<$14,999, $15,000–24,999, $25,000–49,999, $50,000–74,999, ≥$75,000), dependents in the household (yes/no, where those with missing data were assumed to have no children), employed (yes/no, where positive responses to employed for wages or self‐employed were considered employed), current age group (18–24, 25–29, 30–34, 35–39, 40–44, 45–49, 50–54, 55–59, 60–64), and marital status (yes/no, where positive responses to married or separated were coded as married). Proportion of unemployed state residents by year was included to address time‐varying confounding by economic conditions. These economic data for 2010–2018 were obtained from the University of Kentucky Poverty Center's National Welfare Dataset^23^ while comparable data from 2020 were from the Bureau of Labor Statistics.^24^ Specific questions and response types for all variables are included in the Supplementary Appendix (Table S3).

### 2.3 Statistical analysis

Descriptive analyses included the unadjusted percent (%) for each outcome, overall and stratified by expansion status. We also graphed the survey‐weighted percent of those with guideline‐adherent cervical cancer screening over time by race/ethnicity.

To estimate changes in AIAN and then White guideline‐adherent cervical cancer screening before and after the Medicaid expansions, we used difference‐in‐differences (DID) regression. This study design compares changes in the prevalence of guideline‐adherent cervical cancer screening before and after the Medicaid expansions (difference 1) between those residing in expansion and non‐expansion states (difference 2). The primary, fully adjusted DID model is:

$$\begin{array}{cc} Y_{\mathrm{ist}}= & \;\beta_{0}+\beta_{1}\left({\text{Expansion}}_{s}\times {\text{Post}}_{y}\right)+\beta_{2}{\text{Year}}_{t}^{\prime }+\beta_{3}{\text{State}}_{s}^{\prime }\\ & \;+\beta_{4}X_{\mathrm{ist}}^{\prime }+\beta_{5}Z_{\mathrm{st}}+\varepsilon_{\mathrm{ist}} \end{array}$$

where $Y_{\mathrm{ist}}$ is a binary outcome for person *i* in state *s* and year *t*. Expansion and Post are 0/1 indicators of whether a person lives in an expansion state or not and if the outcome is measured pre‐ or post‐expansion, respectively. ${\text{Year}}_{t}$ and ${\text{State}}_{s}$ are vectors of year and state fixed effects, $X_{\mathrm{ist}}$ is a vector of individual level covariates (age, income, education, dependent children, marital status, working status), and $Z_{\mathrm{st}}$ is a state's yearly proportion unemployed. The $\beta_{1}$ coefficient is the DID estimator.

We also used an event study design as a robustness check to investigate the impact of the staggered timing of the Medicaid expansions. The event study models also allow us to detect effects that might take time to observe and assess the parallel trends assumption that is foundational to DID. To do so, we created a variable measuring relative time since Medicaid expansion to accommodate expansions in different years, although 27 of the 32 expansion states did so in 2014. The beta coefficients for the interactions between expansion status and the relative time indicators, when compared to the year prior to expansion, measure if trends in the outcome increased or decreased between exposed and unexposed states (Figures S2 and S3). We used a joint test of whether pre‐expansion interaction terms were equal to zero as a parallel trends assessment (Table S4).

Regression models were linear probability models, with betas multiplied by 100 to be interpreted as percentage point changes in the outcome. Models were estimated without sampling weights.^25^ We reported results from the fully adjusted models (Figure 2) and provided output from the minimally adjusted models (Table S5).

### 2.4 Sensitivity analyses

First, we excluded states that expanded Medicaid to low‐income adults prior to 2014 (DC, DE, MA, NY, VT). Second, we excluded people pregnant at the time of the interview. Third, to determine if those interviewed within 365 days of the expansion may be attenuating the association, we excluded those interviewed during a 365‐day washout period (Figure S5). Fourth, we applied the BRFSS survey weights. Fifth, we excluded those ages 18–24 and sixth, we excluded those ages 45–64 to focus on experiences of reproductive aged people. Lastly, because many AIAN people may identify as Hispanic or as multiracial, we repeated the DID analysis among those that identified AIAN to be their preferred racial category, which increased the available analytic sample size to 5974 people.

Analyses were performed using Stata MP, version 14.2,^26^ and package reghdfe.^27^ This research was approved by our university's IRB (#00002645).

## 3 RESULTS

### 3.1 Descriptive analyses

**FIGURE 1:**
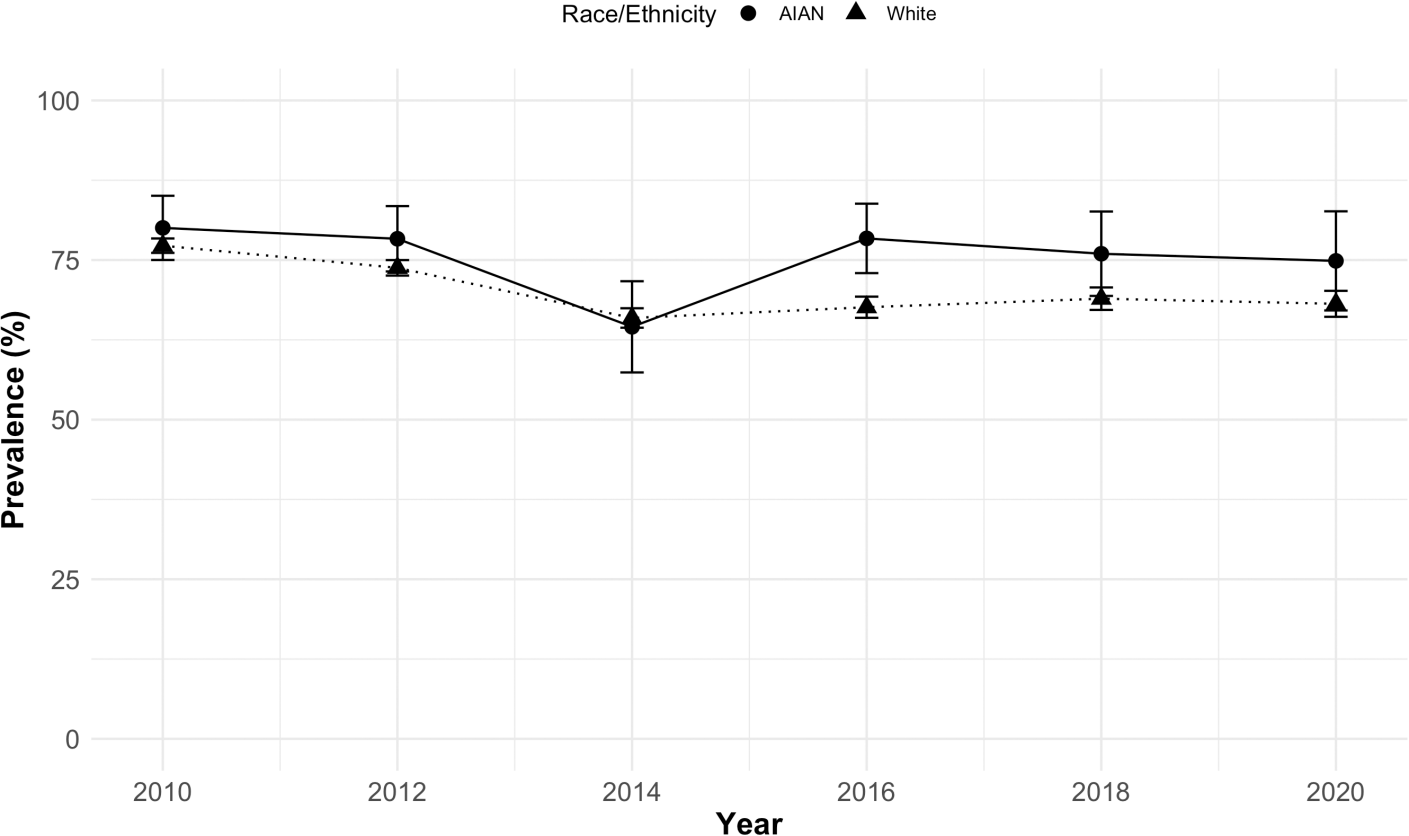
Trends in guideline‐adherent cervical cancer screening among Medicaid eligible AIAN and White women by year, BRFSS 2010–2020. The figure shows the prevalence of guideline‐adherent cervical cancer screening by year among US residents ages 18–64 that are likely Medicaid eligible. AIAN women are represented by the circles and solid line and White women by the triangles and dashed line. BRFSS provided survey weights were applied to calculate prevalence values. The *x*‐axis is survey year and the *y*‐axis guideline‐adherent cervical cancer screening prevalence. AIAN, American Indian and Alaska Natives; BRFSS, Behavioral Risk Factor Surveillance System.

Pooled over time, the AIAN unweighted prevalence of guideline‐adherent cervical cancer screening was lower among those in Medicaid expansion than non‐expansion states (70.3% vs. 72.9%), while the pooled prevalence among White people was higher in Medicaid expansion states (67.6% vs. 63.7%) (Table 1). In most years (excluding 2016), guideline‐adherent cervical cancer screening prevalence between Medicaid eligible AIAN and White people was comparable (Figure 1).

**TABLE 1:** Descriptive statistics (%) for analytic sample by race/ethnicity, overall, and by expansion status, BRFSS, 2010–2020

	American Indian/Alaska Native						White					
	All	(%)	Expansion	(%)	Non‐expansion	(%)	All	(%)	Expansion	(%)	Non‐expansion	(%)
Outcomes	4753	(100)	3237	(100)	1516	(100)	58,207	(100)	42,363	(100)	15,844	(100)
Guideline‐adherent cervical cancer screening	3382	(71.2)	2277	(70.3)	1105	(72.9)	38,731	(66.5)	28,640	(67.6)	10,091	(63.7)
Missing	625	(13.1)	463	(14.3)	162	(10.7)	6414	(11.0)	4718	(11.1)	1696	(10.7)
Ever had a pap test	4277	(90.0)	2873	(88.8)	1404	(92.6)	53,973	(92.7)	39,238	(92.6)	14,735	(93.0)
Missing	18	(0.4)	10	(0.3)	8	(0.5)	186	(0.3)	128	(0.3)	58	(0.4)
Pap test in the last year	2197	(46.2)	1450	(44.8)	747	(49.3)	24,079	(41.4)	17,866	(42.2)	6213	(39.2)
Missing	523	(11.0)	394	(12.2)	129	(8.5)	4701	(8.1)	3460	(8.2)	1241	(7.8)
Pap test in the last 3 years	3481	(73.2)	2344	(72.4)	1137	(75.0)	40,151	(69.0)	29,656	(70.0)	10,495	(66.2)
Missing	523	(11.0)	394	(12.2)	129	(8.5)	4701	(8.1)	3460	(8.2)	1241	(7.8)
Pap test in the last 5 years	3752	(78.9)	2519	(77.8)	1233	(81.3)	44,620	(76.7)	32,867	(77.6)	11,753	(74.2)
Missing	523	(11.0)	394	(12.2)	129	(8.5)	4701	(8.1)	3460	(8.2)	1241	(7.8)
Has a health plan	3856	(81.1)	2666	(82.4)	1190	(78.5)	43,041	(73.9)	33,054	(78.0)	9987	(63.0)
Missing	12	(0.3)	8	(0.2)	4	(0.3)	194	(0.3)	129	(0.3)	65	(0.4)
Avoided care due to cost	1171	(24.6)	749	(23.1)	422	(27.8)	18,630	(32.0)	12,106	(28.6)	6524	(41.2)
Missing	15	(0.3)	12	(0.4)	3	(0.2)	158	(0.3)	120	(0.3)	38	(0.2)
Had a checkup in the last year	3229	(67.9)	2178	(67.3)	1051	(69.3)	35,763	(61.4)	26,692	(63.0)	9071	(57.3)
Missing	79	(1.7)	50	(1.5)	29	(1.9)	967	(1.7)	682	(1.6)	285	(1.8)
Covariates												
Is married	1451	(30.5)	1015	(31.4)	436	(28.8)	20,242	(34.8)	14,326	(33.8)	5916	(37.3)
Missing	42	(0.9)	26	(0.8)	16	(1.1)	291	(0.5)	212	(0.5)	79	(0.5)
Is employed	1777	(37.4)	1220	(37.7)	557	(36.7)	21,976	(37.8)	16,207	(38.3)	5769	(36.4)
Missing	30	(0.6)	24	(0.7)	6	(0.4)	222	(0.4)	161	(0.4)	61	(0.4)
Has dependent(s)	3333	(70.1)	2236	(69.1)	1097	(72.4)	31,387	(53.9)	22,552	(53.2)	8835	(55.8)
Missing	0	(0.0)	0	(0.0)	0	(0.0)	0	(0.0)	0	(0.0)	0	(0.0)
Educational attainment												
<HS grad/GED	983	(20.7)	613	(18.9)	370	(24.4)	7445	(12.8)	4873	(11.5)	2572	(16.2)
HS grad/GED	1857	(39.1)	1339	(41.4)	518	(34.2)	22,038	(37.9)	16,052	(37.9)	5986	(37.8)
Some college/tech	1468	(30.9)	984	(30.4)	484	(31.9)	19,913	(34.2)	14,654	(34.6)	5259	(33.2)
College grad	444	(9.3)	301	(9.3)	143	(9.4)	8769	(15.1)	6756	(15.9)	2013	(12.7)
Missing	1	(0.0)	0	(0.0)	1	(0.1)	42	(0.1)	28	(0.1)	14	(0.1)
Household income												
<15K	2651	(55.8)	1787	(55.2)	864	(57.0)	32,239	(55.4)	23,517	(55.5)	8722	(55.0)
$15–25K	1724	(36.3)	1174	(36.3)	550	(36.3)	22,329	(38.4)	16,090	(38.0)	6239	(39.4)
$25–50K	378	(8.0)	276	(8.5)	102	(6.7)	3637	(6.2)	2754	(6.5)	883	(5.6)
$50–75K	0	(0.0)	0	(0.0)	0	(0.0)	1	(0.0)	1	(0.0)	0	(0.0)
$75K+	0	(0.0)	0	(0.0)	0	(0.0)	1	(0.0)	1	(0.0)	0	(0.0)
Missing	0	(0.0)	0	(0.0)	0	(0.0)	0	(0.0)	0	(0.0)	0	(0.0)
Age group												
18–24	471	(9.9)	331	(10.2)	140	(9.2)	6212	(10.7)	4545	(10.7)	1667	(10.5)
25–29	529	(11.1)	347	(10.7)	182	(12.0)	5831	(10.0)	4151	(9.8)	1680	(10.6)
30–34	608	(12.8)	406	(12.5)	202	(13.3)	6660	(11.4)	4741	(11.2)	1919	(12.1)
35–39	597	(12.6)	400	(12.4)	197	(13.0)	6445	(11.1)	4624	(10.9)	1821	(11.5)
40–44	499	(10.5)	320	(9.9)	179	(11.8)	5844	(10.0)	4255	(10.0)	1589	(10.0)
45–49	496	(10.4)	350	(10.8)	146	(9.6)	6060	(10.4)	4439	(10.5)	1621	(10.2)
50–54	549	(11.6)	382	(11.8)	167	(11.0)	6851	(11.8)	5066	(12.0)	1785	(11.3)
55–59	561	(11.8)	404	(12.5)	157	(10.4)	7332	(12.6)	5404	(12.8)	1928	(12.2)
60–64	443	(9.3)	297	(9.2)	146	(9.6)	6972	(12.0)	5138	(12.1)	1834	(11.6)
Missing	0	(0.0)	0	(0.0)	0	(0.0)	0	(0.0)	0	(0.0)	0	(0.0)

Abbreviation: BRFSS, Behavioral Risk Factor Surveillance System.

### 3.2 Difference‐in‐differences

**FIGURE 2:**
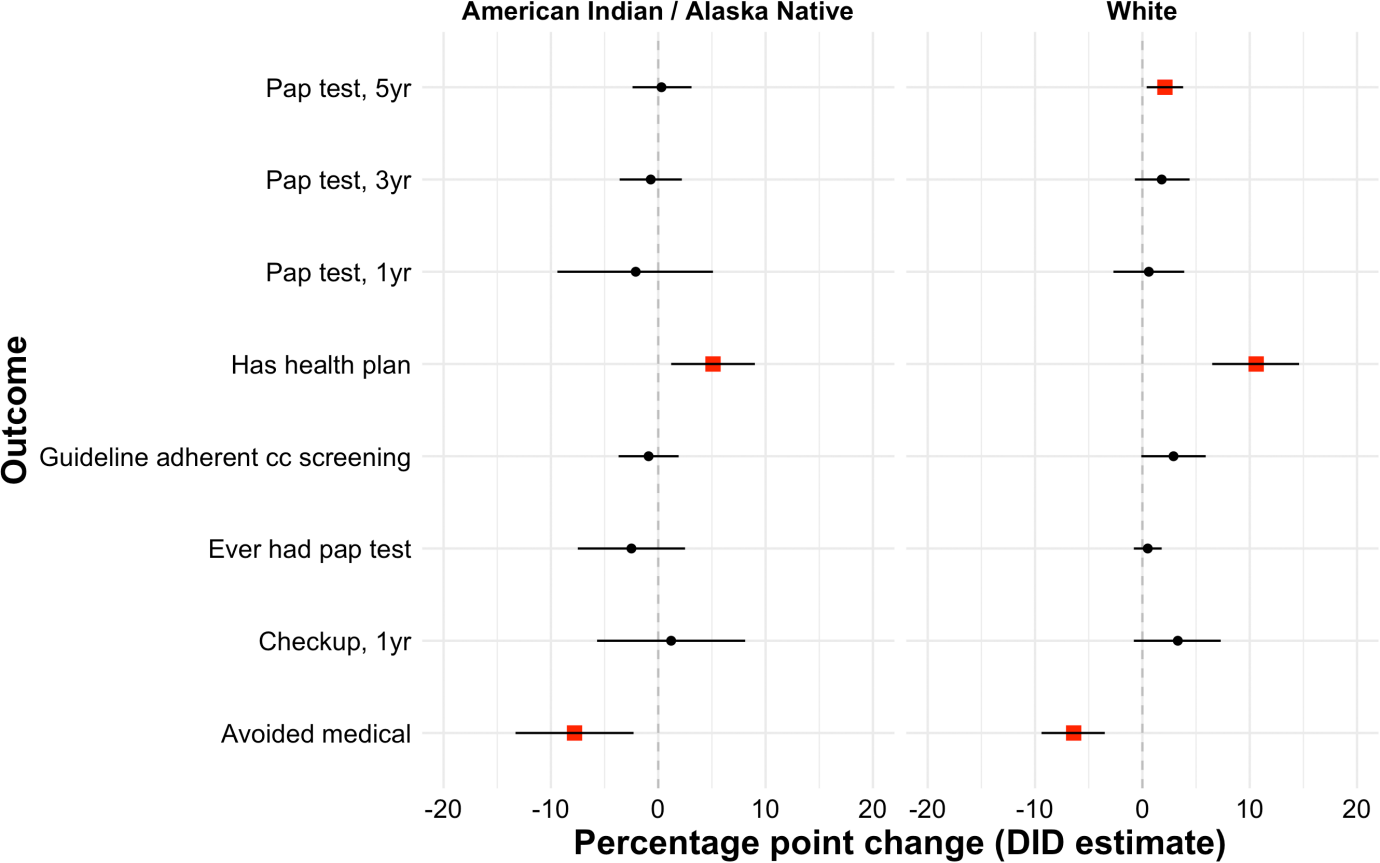
Difference‐in‐differences estimators by study outcome and race/ethnicity, Behavioral Risk Factor Surveillance System, 2010–2020. All DID models included main effects for expansion status and a post expansion indicator (and their interaction), were adjusted for state and year‐specific unemployment rates (%) as well as individual level age group, income, education, dependent children, marital status, working status and included state and year fixed effects. Standard errors were clustered by state. Interaction term beta coefficients (interaction between expansion status and post expansion timeframe) were multiplied by 100 and are indicated by black circles (non‐significant) or red squares (*p* < 0.05) and their 95% confidence intervals by horizontal lines. Negative numbers indicate that expansion states experienced, on average, a decrease in the outcome compared to non‐expansion states, whereas positive numbers indicate that expansion states, on average, experienced an increase in the outcome compared to non‐expansion states. Due to missingness in outcome values, sample sizes for each outcome differ. cc, cervical cancer; DID, difference‐in‐differences; yr, year.

Among AIAN people, residing in an expansion state was associated with a 5‐percentage point (ppt) increase (95% confidence interval [CI]: 1, 9 ppts) in having a health plan and an 8 ppt decrease (95% CI: −13, −2 ppts) in avoiding medical care due to cost, after the Medicaid expansions. However, guideline‐adherent cervical cancer screening decreased by 1 ppt (95% CI: −4, 2 ppts) among expansions states, though the 95% CI includes the null value. Estimates for the other outcomes among the AIAN sample included the null value (Figure 2).

Among White people, residing in an expansion state was associated with an 11 ppt increase (95% CI: 7, 15 ppts) in having a health plan and a 6 ppt decrease (95% CI: −9, −4) in avoiding medical care due to cost. Guideline‐adherent cervical cancer screening increased 3 ppts (95% CI: −0, 6 ppts), though the 95% CI included the null value. Furthermore, the Medicaid expansions were associated with a 2 ppt increase (95% CI: 0, 4 ppt) in having had a pap test in the last 5 years. The remaining outcomes had 95% CIs that contained the null value.

### 3.3 Robustness check and sensitivity analyses

A Wald test of the pre‐expansion beta coefficients from the event study models indicate the parallel trends assumption may be violated for the White sample, particularly for the outcomes of having had a pap test in the last 3 years and the last 5 years (Table S4). The event study models also indicate sustained increases in healthcare coverage and decreases in avoiding care due to costs among White people immediately following the expansions (Figure S3). Results were not consistent in the AIAN‐specific event study models where the increase in healthcare coverage was not immediate, nor sustained (Figure S4). Similarly, there was an immediate decrease in those that avoided care due to cost, but the decrease was not sustained.

Among AIAN people, we observed some novel results in the sensitivity analyses (Table S6). First, when excluding pregnant people or those ages 45–64 years, all associations attenuated. Second, when we applied survey weights, the expansions became associated with a 11 ppt decrease in having a checkup in the last year (95% CI: −21, −7). Among White people, we observed that the results were largely consistent across sensitivity analyses. When excluding early expanding states, the Medicaid expansions were also associated with a 4 ppt increase (95% CI: 0, 8 ppts) in having had a checkup in the last year. When applying survey weights, the association with having had a pap test in the last 5 years was attenuated. Lastly, when using the preferred race variable to identify AIAN people, the relationship between the expansions and avoiding medical care due to cost, attenuated and became non‐significant.

## 4 DISCUSSION

We hypothesized that the ACA's Medicaid expansion would be associated with increases in guideline‐adherent cervical cancer screening, particularly among AIAN, due to increased insurance coverage and subsequent health care utilization, as well as potential changes to IHS funding structures. Our results suggest that the Medicaid expansions were associated with improvements in insurance coverage and engagement in needed care without worrying about associated costs. However, across each definition of cervical cancer screening, we did not observe improvements in screening. The increase in Medicaid coverage may not have translated into changes in healthcare use, particularly for AIAN cervical cancer screening.

A recent systematic review of the impact of the Medicaid expansions on cancer outcomes reported seven studies with increases in cervical cancer screening, four decreases in screening, and two concluded there was no change.^28^ Our results of no changes in screening are consistent with a study using BRFSS data.^29^ However, Tummalapalli and Keyhani used data through 2017, and it is possible that it takes time for an impact to occur. Indeed, we estimated that among White people in expansion states, there was an increase in having had a pap test in the last 5 years following the Medicaid expansions, though results from the event study model suggests the DID model might be biased. Hendryx and Luo used BRFSS data and observed an increase in cervical cancer screening following the Medicaid Expansions.^30^ However, their outcomes were not guideline‐adherent measures and included limited pre‐ and post‐expansion data.

There are several reasons why the Medicaid expansions may not associate with increases in guideline‐adherent cervical cancer screening. The National Breast and Cervical Cancer Early Detection Program (NBCCEDP) was created in 1990 to increase screening among low‐income people without health insurance coverage. The NBCCEDP may play an important role in supporting screening,^31^ as Alharbi et al. reported similar results and demonstrate that low‐income people with Medicaid were less likely to receive preventative services like pap tests, suggesting there might be something about Medicaid itself that provides a barrier to screening. BRFSS estimates guideline‐adherent screening to be 65%–80% among AIAN and White people, depending on the year, which may be too high to see gains from increased insurance coverage, particularly if coverage does not overcome other barriers to screening like access to transportation, distance to services, childcare coordination, or availability of healthcare, etc.^32^, ^33^, ^34^ There are also ways that the Medicaid expansions may ultimately work to decrease cervical cancer related morbidity and mortality, which we were unable to examine. The expansions could increase follow up after a positive screen by decreasing perceived costs of treatment or could decrease the age at which women enter into regular well‐women visits.

Our results may suggest that the Medicaid expansions are associated with some measures of cervical cancer screening uptake for White but not AIAN people, as indicated by the positive association for having had a pap test in the last 5 years and the near significant positive association with guideline‐adherent cervical cancer screening. Though, of note, we rejected the null hypothesis of parallel trends in the outcome of having had a pap test in the last 5 years (Table S4) and the recall period includes some pre‐expansion time. Further, we are unable to determine if the lack of association in cervical cancer screening among AIAN people are related to estimate imprecision.

### 4.1 Strengths

We make an important contribution by focusing on screening and healthcare coverage among AIAN people, a group often excluded from research, using a nationally representative dataset. We also operationalized the cervical cancer screening outcome using up‐to‐date guidelines. Lastly, our study includes up to 5 years of post‐expansions data, enabling us to observe delayed impacts, if there are impacts to observe.

### 4.2 Limitations

Our research was limited by data inadequacies. The BRFSS does not regularly collect information about tribal affiliation, so we are unable to address AIAN heterogeneity. This practice of aggregating AIAN into a single group can be problematic, yet, suppressing AIAN data represents its own problematic practice.^35^ Second, it is unclear the extent to which the AIAN sampled in the BRFSS adequately represent the diversity in experience of AIAN people. Further, because the CDC does not prioritize AIAN status in creating racial/ethnic categories for analyses, we could not create an AIAN group inclusive of those identifying as AIAN in combination with other racial or ethnic identities. However, our sensitivity analysis that used AIAN preferred race, did not result in an alteration of the relationship between Medicaid expansion and screening.

A second set of limitations relate to measurement error. First, there was discord between the age groupings in BRFSS and guideline adherence recommendations. Because we assigned the 18–24‐year‐olds to the “no screening” needed to be guideline‐adherent group, we are misclassifying those 22–24 years of age as not guideline adherent, even if they are. Second, some of the recall periods for screening measures are long and include pre‐expansion time. Third, some individuals are missing information on the number of adults in their household, one variable we used to determine Medicaid eligibility. Those with missing values were assigned 1, meaning we likely underestimated the number of people at or below 138% FPL and misclassified some as Medicaid ineligible when they are eligible. The situation is similar regarding the number of children in a household. Fourth, there is potential for exposure misclassification in the event study models found in supplemental materials. We assigned relative time by survey year (e.g., 2010, 2014) and some states expanded part‐way through a survey year. Lastly, we conducted an intention to treat analysis because not all people eligible for Medicaid would have enrolled and not all of those identifying as AIAN qualify for IHS, which is one mechanism by which the expansions might impact screening. Of note, there is anecdotal evidence that some AIAN people may not enroll in Medicaid because they interpret it as a violation of the Federal Trust Responsibility.^5^, ^9^

## 5 CONCLUSIONS

Amidst ongoing discussions for its repeal, the impacts of the ACA on health care use are still being investigating. While often not framed in this way, the ACA could be thought of as one way that the U.S. federal government can uphold its Trust Responsibility to Native nations. With this understanding, we can look at the impacts of the ACA as being obligated to impact the health of AIAN people. Yet, answering this question of positive impact is challenging given ongoing limitations—irrelevance, quality, and external control—of available data.^36^ While we observed improvements in health care coverage and avoiding care, we did not observe changes to guideline‐adherent cervical cancer screening among AIAN following the Medicaid expansions. Given the ongoing disproportionate burden of cervical cancer among AIAN people, improving cervical cancer screening uptake and delivery should be prioritized to reduce preventable deaths.

## AUTHOR CONTRIBUTIONS

**Danielle R. Gartner:** Conceptualization (equal); formal analysis (lead); funding acquisition (equal); methodology (equal); visualization (lead); writing – original draft (lead); writing – review and editing (equal). **Jessica Y. Islam:** Conceptualization (equal); methodology (equal); writing – review and editing (equal). **Claire E. Margerison:** Conceptualization (equal); funding acquisition (equal); methodology (equal); writing – review and editing (equal).

## CONFLICT OF INTEREST

Authors have no conflicts of interest to declare.

## ETHICS APPROVAL STATEMENT

This research was approved by the lead author's university IRB (#00002645).

## Supporting information


Data S1.

## Data Availability

The Behavioral Risk Factor Surveillance data used for this project are publicly available for download at a website maintained by Centers for Disease Control and Prevention.
