# Supplementary material for: Medicaid expansions and differences in guideline‐adherent cervical cancer screening between American Indian and White women
Source: Cancer Med. 2023 Jan 11;12(7):8700–9. doi: 10.1002/cam4.5593 (PMC10134301; doi:10.1002/cam4.5593)
Supplement: Supplementary file 1 — Data S1. [file CAM4-12-8700-s001.docx]

**APPENDIX**

**Appendix Figure 1**: American Indian & Alaska Native study population and sample, BRFSS, 2010-2020

Likely eligible for Medicaid

N=6,415

No hysterectomy

N=4,754

Valid income & household size

N=14,742

Income > 138% FPL

50 states + DC

N=17,445

Residing in territories

Missing income or household size

BRFSS 2010-20

N=2,717,123

AIAN Females

N=23,407

Ages 18-64

N=17,445

Males, non-AIAN

<18 or >44

Had hysterectomy or missing

hysterectomy status

Non-missing covariates

Eligible for analyses

N=4,681

Missing any covariate

Missing outcome value

(differs by outcome)

**Appendix Table 1:** Analytic sample sizes by outcome and race/ethnicity

| **Outcome** | **Race/ethnicity** ^a^ | **Sample Size** |
| --- | --- | --- |
| Guideline-adherent cervical cancer screening | AIAN | 4084 |
| Ever had a pap test | AIAN | 4678 |
| Pap test in the last year | AIAN | 4185 |
| Pap test in the last 3 years | AIAN | 4185 |
| Pap test in the last 5 years | AIAN | 4185 |
| Has a health plan | AIAN | 4669 |
| Avoided care due to cost | AIAN | 4666 |
| Had a checkup in the last year | AIAN | 4607 |
| Guideline-adherent cervical cancer screening | White | 51445 |
| Ever had a pap test | White | 57597 |
| Pap test in the last year | White | 53146 |
| Pap test in the last 3 years | White | 53146 |
| Pap test in the last 5 years | White | 53146 |
| Has a health plan | White | 57471 |
| Avoided care due to cost | White | 57507 |
| Had a checkup in the last year | White | 56713 |

^a^ AIAN = American Indian & Alaska Native

**Appendix Table 2**: Medicaid expansion dates, states, and timeframes for pre-expansion, post-expansion, and 0-365 days post expansion

| **Expansion Date** | **States** | **Pre-expansion** | **1 to 365 days post expansion ^a^** | **More than 1 year post expansion** |
| --- | --- | --- | --- | --- |
| 1/1/2014 | Arizona, Arkansas, California, Colorado, Connecticut, Delaware, District of Columbia, Hawaii, Illinois, Iowa, Kentucky, Maryland, Massachusetts, Minnesota, Nevada, New Jersey, New Mexico, New York, North Dakota, Ohio, Oregon, Rhode Island, Vermont, Washington, West Virginia | 1/1/2010 – 12/31/2013 | 1/1/2014 – 12/31/2014 | 1/1/2015 – 12/31/2021 |
| Non expansion | Alabama, Florida, Georgia, Kansas, Mississippi, Missouri, North Carolina, Oklahoma, South Carolina, South Dakota, Tennessee, Texas, Wisconsin, Wyoming | 1/1/2010 – 12/31/2013 | 1/1/2014 – 12/31/2014 | 1/1/2015 – 12/31/2021 |
| 4/1/2014 | Michigan | 1/1/2010 – 3/31/2014 | 4/1/2014 – 3/31/2015 | 4/1/2015 – 12/31/2021 |
| 8/15/2014 | New Hampshire | 1/1/2010 – 8/14/2014 | 8/15/2014 – 8/14/2015 | 8/15/2015 – 12/31/2021 |
| 1/1/2015 | Pennsylvania | 1/1/2010 – 12/31/2014 | 1/1/2015 – 12/31/2015 | 1/1/2016 – 12/31/2021 |
| 2/1/2015 | Indiana | 1/1/2010 – 1/31/2015 | 2/1/2015 – 1/31/2016 | 2/1/2016 – 12/31/2021 |
| 9/1/2015 | Alaska | 1/1/2010 – 8/31/2015 | 9/1/2015 – 8/31/2016 | 9/1/2016 – 12/31/2021 |
| 1/1/2016 | Montana | 1/1/2010 – 12/31/2015 | 1/1/2016 – 12/31/2016 | 1/1/2017 – 12/31/2021 |
| 7/1/2016 | Louisiana | 1/1/2010 – 6/30/2016 | 7/1/2016 – 6/30/2017 | 7/1/2017 – 12/31/2021 |
| 1/1/2019 | Virginia | 1/1/2010 – 12/31/2018 | 1/1/2019 – 12/31/2019 | 1/1/2020 – 12/31/2021 |
| 1/10/2019 | Maine | 1/1/2010 – 1/9/2019 | 1/10/2019 – 1/9/2020 | 1/10/2020 – 12/31/2021 |
| 1/1/2020 | Idaho, Utah | 1/1/2010 – 12/31/2019 | 1/1/2020 – 12/31/2020 | 1/1/2021 – 12/31/2021 |
| 10/1/2020 | Nebraska | 1/1/2010 – 9/30/2020 | 10/1/2020 – 9/30/2021 | 10/1/2021 – 12/31/2021 |

^a^ Those interviewed during the washout period (0-365 days post expansion) were considered untreated/unexposed for analyses. However, for the third sensitivity analyses, those interviewed during this period were excluded from analyses.

**Appendix Table 3:** Survey Questions, possible answers, and final function form of study outcomes and covariates

| **Concept** | **Survey Question / Prompt** | **Possible Answers** | **Outcome** | **Final Functional Form** |
| --- | --- | --- | --- | --- |
| **OUTCOMES** | | | | |
| Health care coverage | Do you have any kind of health care coverage, including health insurance, prepaid plans such as HMOs, or government plans such as Medicare, or Indian Health Service? | - Yes - No | Has a health plan | No / Yes |
| Check up in last year | About how long has it been since you last visited a doctor for a routine checkup? [A routine checkup is a general physical exam, not an exam for a specific injury, illness, or condition.] | - Within the past year (< 12 months) - Within the past 2 years (1 year but < 2 years) - Within the past 5 years (2 years but < 5 years) - Five or more years | Had a routine checkup within the past year | No / Yes |
| Ever had a pap smear | Have you ever had a pap test? | - Yes - No | Ever had a pap smear | No / Yes |
| Cervical cancer screening | How long has it been since you had your last Pap test? | - Within the past year (< 12 months) - Within the past 2 years (1 year but < 2 years) - Within the past 3 years (2 years but < 3 years) - Within the past 5 years (3 years but < 5 years) - Five or more years | 2010-2012: Had a pap test in the past 3 years  2014-2018:   - Ages 18-24: No pap testing - Ages 25-29: Had a pap test in the past 3 years - Ages 30-44: Had a pap test AND had an H.P.V. test in the past 5 years OR Had a pap test in the past 3 years | No / Yes |
|  | How long has it been since you had your last H.P.V. test? | - Within the past year (< 12 months) - Within the past 2 years (1 year but < 2 years) - Within the past 3 years (2 years but < 3 years) - Within the past 5 years (3 years but < 5 years) - Five or more years |  |  |
| **COVARIATES** | | | | |
| Education | What is the highest grade or year of school you completed? | - Never attended or only kindergarten - Grades 1-8 (elementary) - Grades 9-11 (some high school) - Grade 12 or GED (high school graduate) - College 1 year to 3 years (Some college or technical school) - College 4 years or more (college graduate) | Highest level of education  [Grouped never attended, elementary and some high school] | Less than high school / High school diploma or GED / Some college / College or more |
| Dependents at home | How many children less than 18 years of age live in your household? | - List number of children | Has dependents in the household  [number of children > 0] | No / Yes |
| Employed | Are you currently...? | - Employed for wages - Self-employed - Out of work for 1 year or more - Out of work for less than 1 year - A homemaker - A student - Retired - Unable to work | Currently working [Employed for wages, self-employed] | No / Yes |
| Marital status | Are you…? | - Married - Divorced - Widowed - Separated - Never married - A member of an unmarried couple | Married  [Currently married or separated] | No / Yes |
| Household Income | Is your annual household income from all sources…? | - < $10,000 - < $15,000 ($10,000 to less than $15,000) - < $20,000 ($15,000 to less than $20,000) - < $25,000 ($20,000 to less than $25,000) - < $35,000 ($25,000 to less than $30,000) - < $50,000 ($35,000 to less than $50,000) - < $75,000 ($50,000 to less than $75,000) - $75,000 or more | Household income and household size were used to restrict the sample to those at or below 138% FPL. Household income was also used as a covariate in multivariable modeling. | less than $14,999 / $15,000-24,999 / $25,000-49,999 |
| **INCLUSION/EXCLUSION CRITERIA** | | | | |
| Sex | 2010-2018: What is your sex? or What was your sex at birth? Was it... | - Male - Female - Don’t know/Not sure - Refused | Female  [Male or Female]  EXCLUDED MALES | No / Yes |
|  | 2020: What was your sex at birth? Was it male or female? |  |  |  |
| 138% Federal Poverty Level (FPL) | Is your annual household income from all sources…? | - < $10,000 - < $15,000 ($10,000 to less than $15,000) - < $20,000 ($15,000 to less than $20,000) - < $25,000 ($20,000 to less than $25,000) - < $35,000 ($25,000 to less than $30,000) - < $50,000 ($35,000 to less than $50,000) - < $75,000 ($50,000 to less than $75,000)   $75,000 or more | Household income and household size (adults + children) were used to restrict the sample to those at or below 138% FPL.  EXCLUDED IF > 138% FPL | State, year, household income, and household size specific estimates |
|  | How many children less than 18 years of age live in your household? | - List number of children |  |  |
|  | Excluding adults living away from home, such as students away at college, how many members of your household, including yourself, are 18 years of age or older? | - List number |  |  |
| Hysterectomy | Have you had a hysterectomy? (A hysterectomy is an operation to remove the uterus (womb).) | - Yes - No - Don’t know/Not sure | Had prior hysterectomy  EXCLUDED IF HAD PRIOR HYSTERECTOMY | No / Yes |
| Age group | Based on current age | - Fourteen level age category   - 18-24   - 25-29   - 30-34   - 35-39   - 40-44   - Etc. | Age group  EXCLUDED IF AGE > 64 | 18-24 / 25-29 / 30-34 / 35-39 / 40-44 / etc. |

**Appendix Figure 2:** Visual representation of time allocations by survey year for 2014, 2015, and 2016 expanders, for event study analyses

**2010-11**

**2016-17**

**2012-13**

**2014-15**

**2020-21**

**2018-19**

**2010-11**

**2016-17**

**2012-13**

**2014-15**

**2020-21**

**2018-19**

**2010-11**

**2014-15**

**2012-13**

**2016-17**

**2018-19**

**2020-21**

**-2**

**(referent)**

**-4**

**-6**

**+6**

**+4**

**+2**

**Expansion**

**2016 Expanders**

**Event Time:**

**2015 Expanders**

**2014 Expanders**

**Appendix Figure 3:** Interaction term beta coefficients and 95% confidence intervals for event study regression by outcome among White people

**A) Guideline-adherent cervical cancer screening**


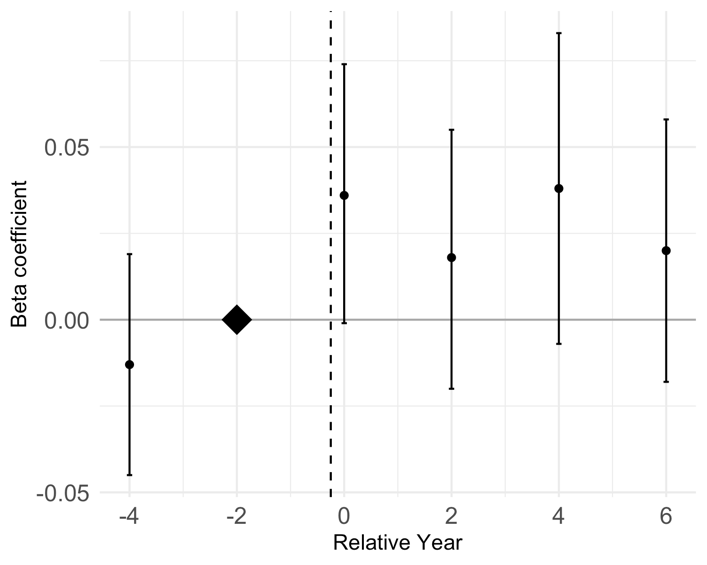


**B) Had a pap smear in the last year**


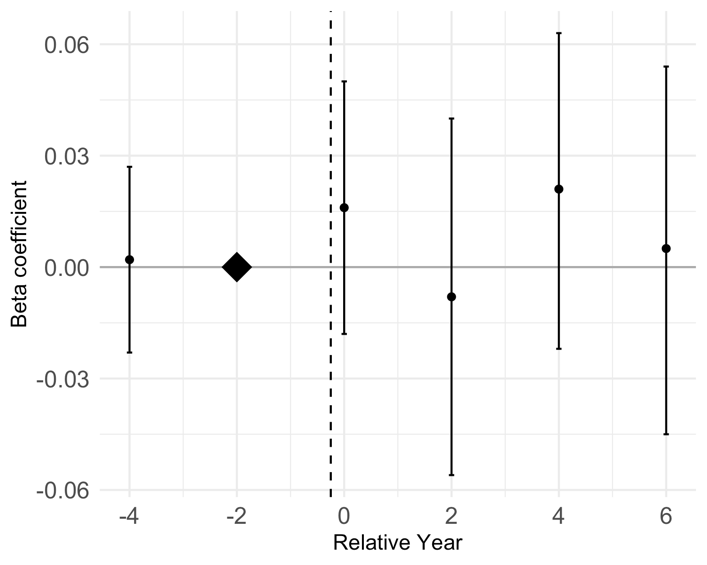


**D) Had a pap smear in the last 5 years**


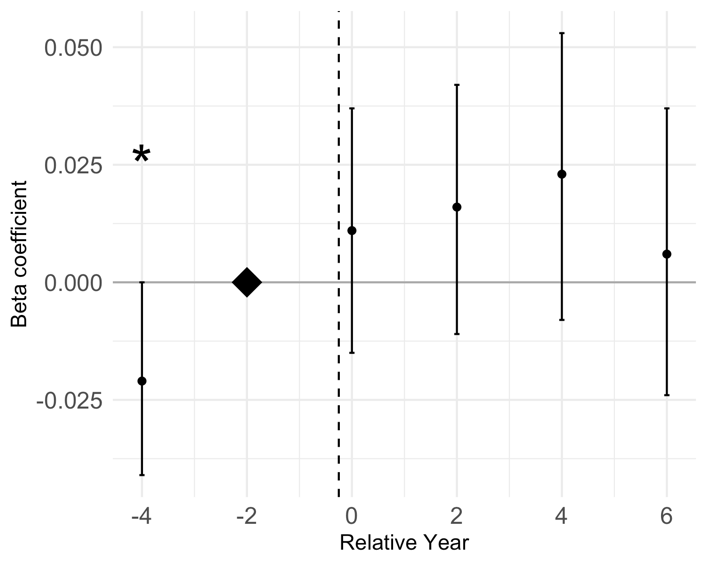


**C) Had a pap smear in the last 3 years**


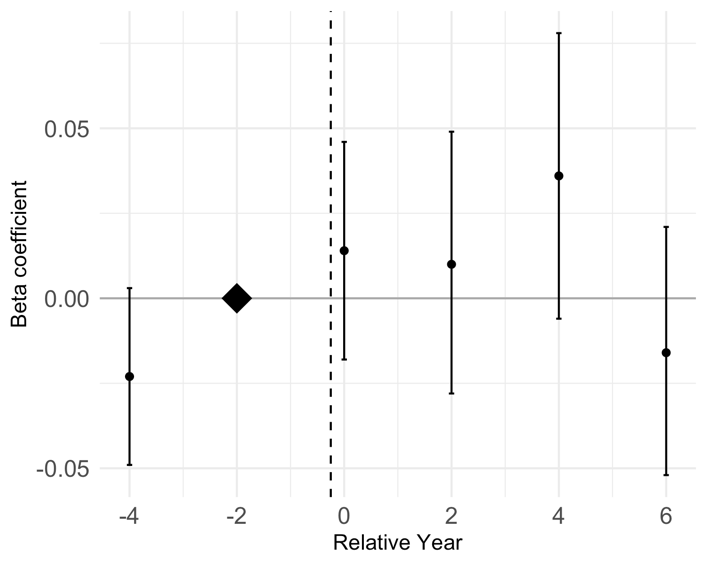


**F) Has health care coverage**


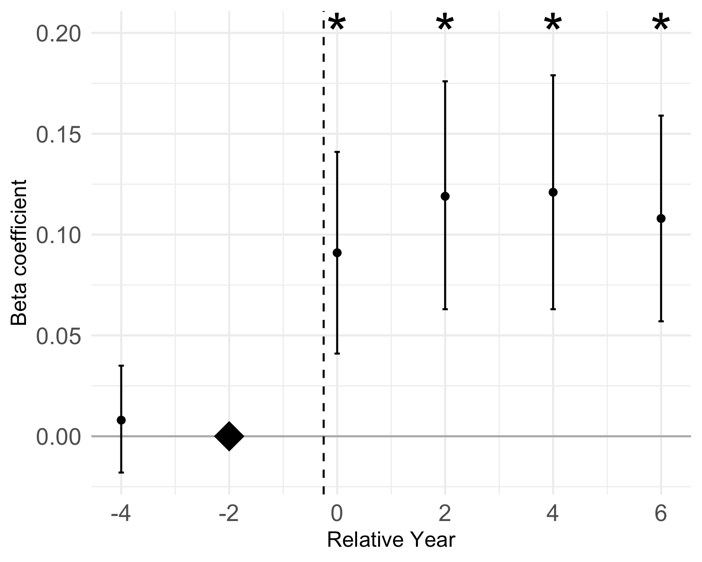


**E) Ever had a pap smear**


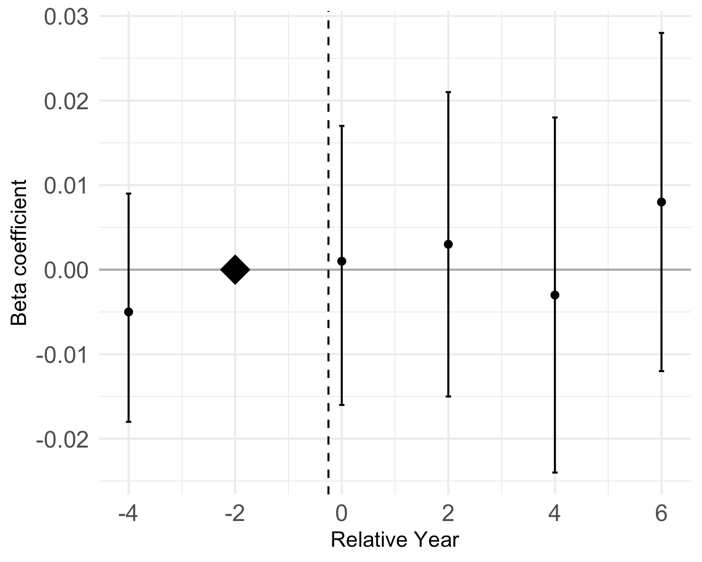


**G) Avoided care because of cost**


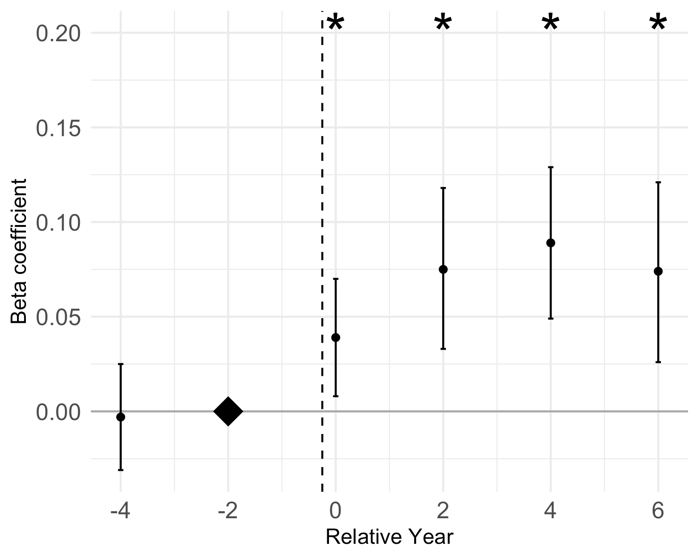


**H) Routine checkup in last year**


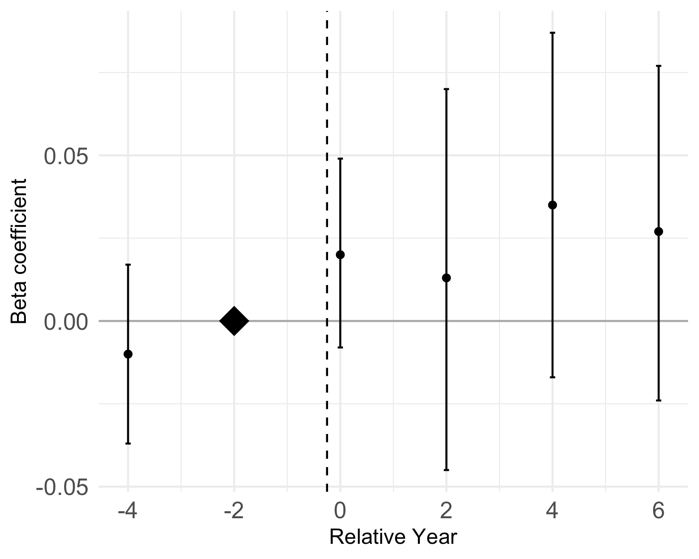


*Legend: All event study models included main effects for relative time and expansion status (and their interaction), were adjusted for state and year-specific unemployment rates (%) as well as individual level age group, income, education, dependent children, marital status, working status and included state and year fixed effects. Standard errors were clustered by state. Interaction term beta coefficients (interaction between relative time and expansion status) are indicated by dots and their 95% confidence intervals by vertical T-bars. The referent was the beta value comparing the outcome between expanded and non-expanded states in the time prior to expansion (Relative Year = -2) and is marked with a shaded diamond. The vertical dotted line indicates the time of the expansion. Asterisks above a data point indicate a significant estimate (p*$\leq$*0.05).* *The model for the outcome of adherence to cervical cancer screening guidelines (A) is at the top left corner, followed by had a pap smear in the last year (B), had a pap smear in the last 3 years (C), had a pap smear in the last 5 years (D), ever had a pap smear (E), have a health plan (F), avoided care because of cost (G), and had a routine checkup in the last year (H).*

**Appendix Figure 4:** Interaction term beta coefficients and 95% confidence intervals for event study regression by outcome among American Indians & Alaska Native people

**B) Had a pap smear in the last year**


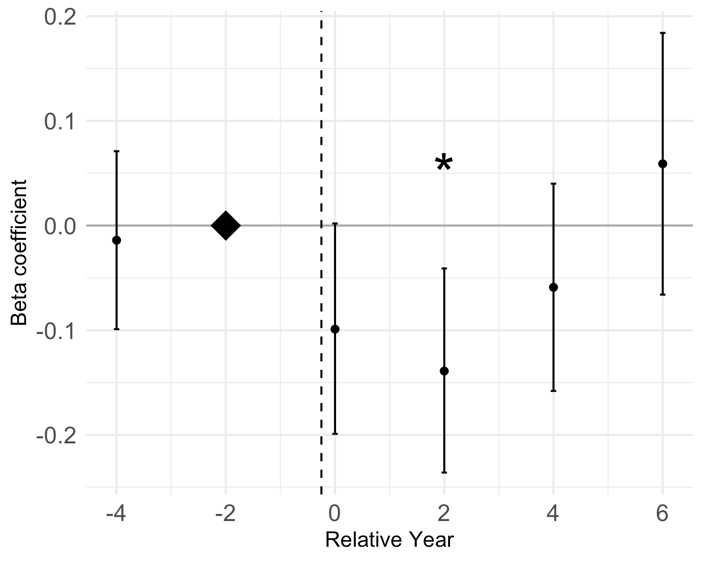


**A) Adherence to cervical cancer screening guidelines**


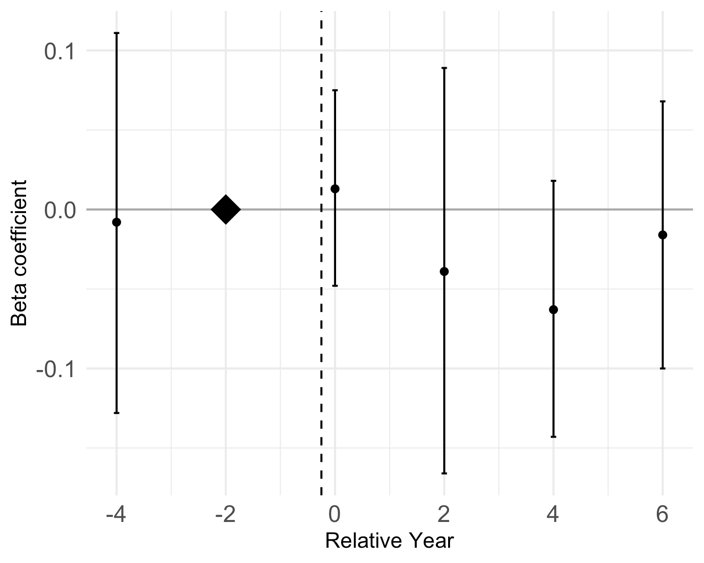


**D) Had a pap smear in the last 5 years**


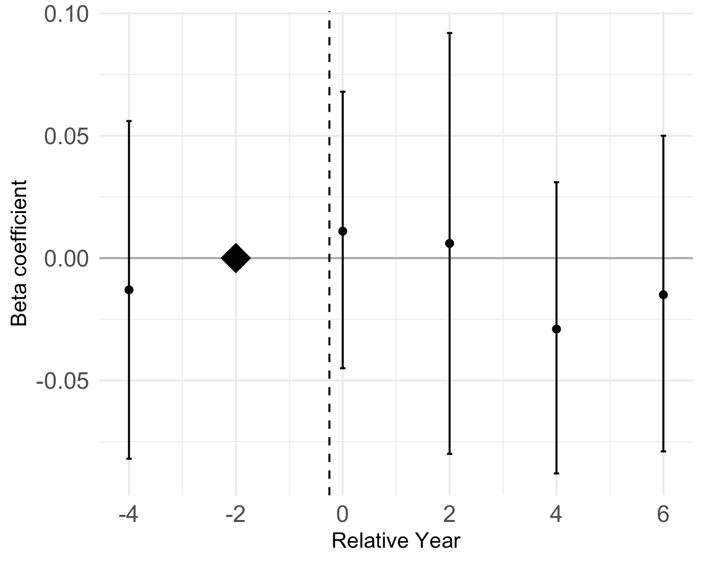


**C) Had a pap smear in the last 3 years**


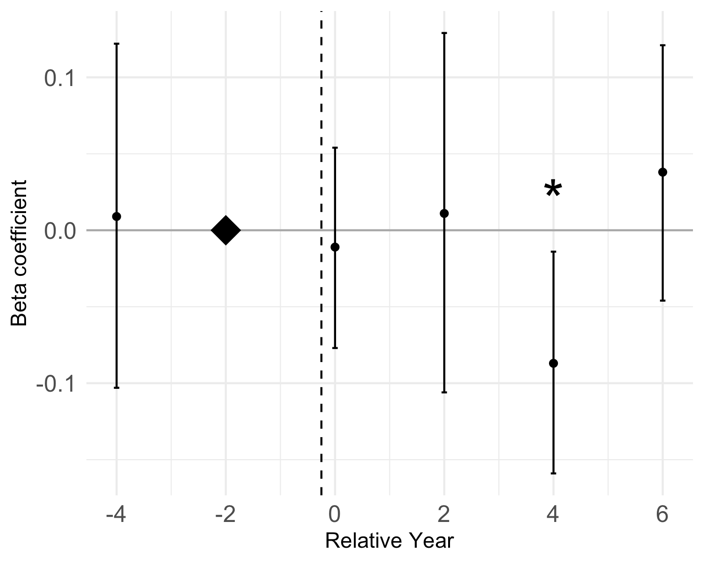


**F) Has health care coverage**


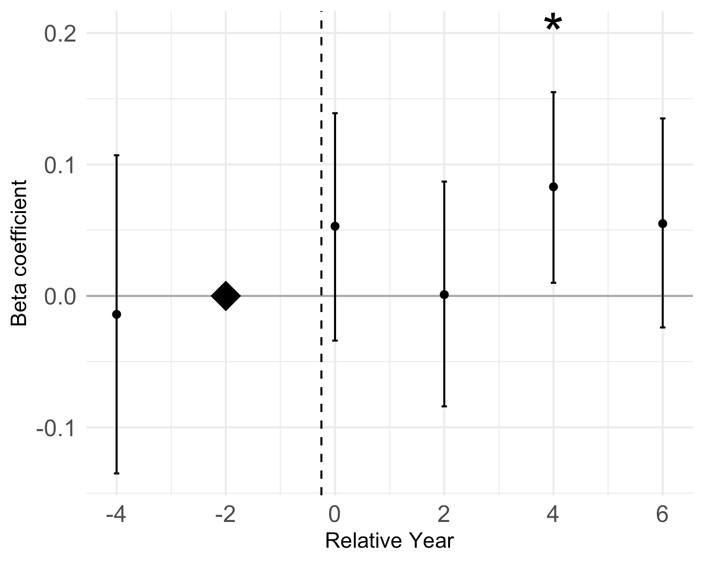


**E) Ever had a pap smear**


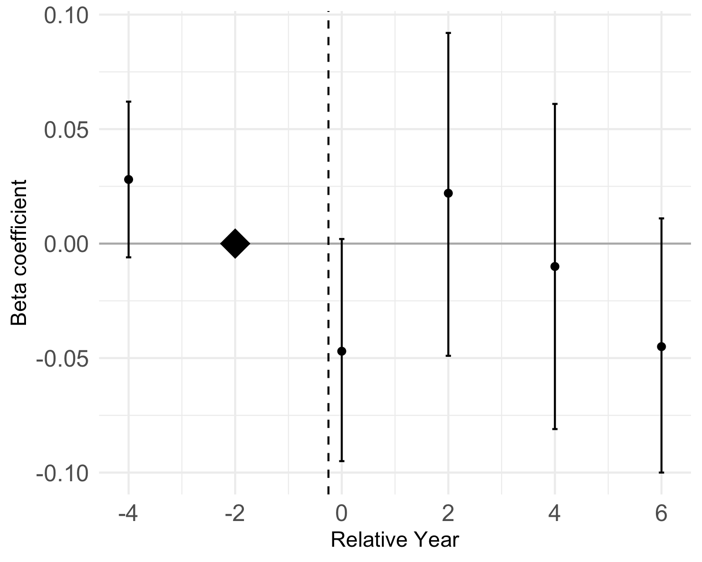


**G) Avoided care because of cost**


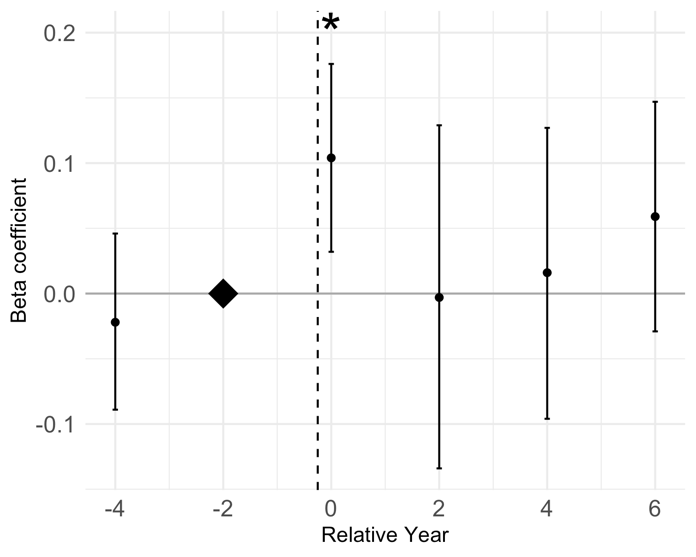


**H) Routine checkup in last year**


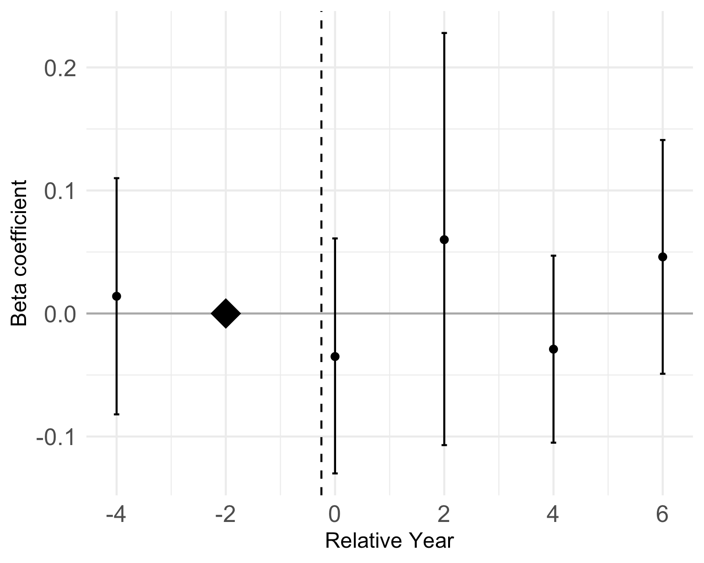


*Legend: All event study models included main effects for relative time and expansion status (and their interaction), were adjusted for state and year-specific unemployment rates (%) as well as individual level age group, income, education, dependent children, marital status, working status and included state and year fixed effects. Standard errors were clustered by state. Interaction term beta coefficients (interaction between relative time and expansion status) are indicated by dots and their 95% confidence intervals by vertical T-bars. The referent was the beta value comparing the outcome between expanded and non-expanded states in the time prior to expansion (Relative Year = -2) and is marked with a shaded diamond. The vertical dotted line indicates the time of the expansion. Asterisks above a data point indicate a significant estimate (p*$\leq$*0.05).* *The model for the outcome of adherence to cervical cancer screening guidelines (A) is at the top left corner, followed by had a pap smear in the last year (B), had a pap smear in the last 3 years (C), had a pap smear in the last 5 years (D), ever had a pap smear (E), have a health plan (F), avoided care because of cost (G), and had a routine checkup in the last year (H).*

**Appendix Table 4**: p-value for Wald test (F-test) that interaction terms between expansion status & relative year are equal prior to Medicaid expansions among American Indian and Alaska Native and White samples

| Outcome | American Indian / Alaska Native | White |  |  |
| --- | --- | --- | --- | --- |
| Guideline-adherent cervical cancer screening | 0.8905 | 0.4197 |  |  |
| Ever had a pap smear | 0.1081 | 0.4782 |  |  |
| Had a pap smear in the last year | 0.7398 | 0.8751 |  |  |
| Had a pap smear in the last 3 years | 0.8670 | **0.0849** |  |  |
| Had a pap smear in the last 5 years | 0.7124 | **0.0467** |  |  |
| Health care coverage | 0.8132 | 0.5237 |  |  |
| Avoided care due to cost | 0.5225 | 0.8253 |  |  |
| Checkup in last year | 0.7715 | 0.4444 |  |  |

Event study regression models included an interaction between indicators for expansion status and the “relative year”, where relative year indicates the timing of the interview date relative to Medicaid expansion in the state of residence. Models also included state and year fixed effects, state and year specific unemployment prevalence, individual level age group, income, education, dependent children, marital status, working status, and standard errors were clustered by state.

Statistical Test: Wald F-test (tests the parallel trends assumption, or the hypothesis that the differences in the outcome, between expansion and control states (i.e., interaction beta coefficients) were the same for each relative year prior to expansion (i.e., all negative years).

P-values <0.10 are bolded (indicate that the beta coefficient differed from zero and provide evidence for rejection of the test’s null hypothesis that trends in the outcome were parallel between expansion and control states prior to expansion).

**Appendix Figure 5:** Visual for (A) main analyses pre-/post-expansion categorization and (B) sensitivity analyses with washout period for difference-in-differences analyses

**A**

For Medicaid expansion states: Interview considered post expansion if the interview date was 365 days or more after the date of expansion. Pre-expansion was any time before that.

For non-expansion states: Interview considered post expansion if interview date was 365 days or more after 1/1/2014. Pre-expansion was any time before that.

For Medicaid expansion states: Interview considered post expansion if the interview date was 365 days or more after the date of expansion. Pre-expansion was any time before the data of expansion.

For non-expansion states: Interview considered post expansion if interview date was 365 days or more after 1/1/2014. Pre-expansion was any time before 1/1/2014.

Washout

**Exclude**

Expansion

2010

2020

Expansion + 365 days

**Pre**

**Post**

**B**

Expansion

2010

2020

Expansion + 365 days

**Pre**

**Post**

**Appendix Table 5:** Difference-in-differences estimators from unadjusted and adjusted regression models of the association between Medicaid expansions and outcomes, BRFSS, 2010-2020

|  | **American Indian & Alaska Native** | | | | | | |  | **White** | | | | | | |  |
| --- | --- | --- | --- | --- | --- | --- | --- | --- | --- | --- | --- | --- | --- | --- | --- | --- |
|  | Minimally Adjusted | | |  | | Fully Adjusted | |  | Minimally Adjusted | |  | | Fully Adjusted | | |  |
| Outcome | B coefficient | (95% CI) |  | | B coefficient | | (95% CI) |  | B coefficient | (95% CI) | |  | | B coefficient | (95% CI) | |
| Guideline-adherent cervical cancer screening | 0.00 | (-0.039, 0.035) |  | | -0.01 | | (-0.037, 0.019) |  | 0.03 | (-0.007, 0.061) | |  | | 0.03 | (-0.001, 0.059) | |
| Ever had a pap test | -0.02 | (-0.074, 0.026) |  | | -0.03 | | (-0.075, 0.025) |  | 0.00 | (-0.011, 0.018) | |  | | 0.01 | (-0.008, 0.018) | |
| Pap test in the last year | -0.03 | (-0.118, 0.069) |  | | -0.02 | | (-0.094, 0.051) |  | 0.00 | (-0.034, 0.038) | |  | | 0.01 | (-0.027, 0.039) | |
| Pap test in the last 3 years | -0.01 | (-0.046, 0.027) |  | | -0.01 | | (-0.036, 0.022) |  | 0.02 | (-0.010, 0.043) | |  | | 0.02 | (-0.007, 0.044) | |
| Pap test in the last 5 years | 0.00 | (-0.035, 0.038) |  | | 0.00 | | (-0.024, 0.031) |  | **0.02** | **(0.003, 0.038)** | |  | | **0.02** | **(0.004, 0.038)** | |
| Has a health plan | **0.05** | **(0.009, 0.089)** |  | | **0.05** | | **(0.012, 0.090)** |  | **0.10** | **(0.063, 0.144)** | |  | | **0.11** | **(0.065, 0.146)** | |
| Avoided care due to cost | -0.07 | (-0.155, 0.007) |  | | **-0.08** | | **(-0.133, -0.023)** |  | **-0.06** | **(-0.093, -0.034)** | |  | | **-0.06** | **(-0.094, -0.035)** | |
| Had a checkup in the last year | 0.01 | (-0.059, 0.080) |  | | 0.01 | | (-0.057, 0.081) |  | 0.03 | (-0.010, 0.072) | |  | | 0.03 | (-0.008, 0.073) | |

CI = confidence interval

Minimally adjusted models include main effects for expansion status and a post expansion indicator (and their interaction) and include state and year fixed effects. Standard errors are clustered by state.

Fully adjusted models include main effects for expansion status and a post expansion indicator (and their interaction) and are adjusted for state and year-specific unemployment rates (%) as well as individual level age group, income, education, dependent children, marital status, working status and included state and year fixed effects. Standard errors are clustered by state.

Estimates from the fully adjusted models are presented in the main text as Figure 2.

Bolded values indicate that the 95% confidence interval crosses the null value of zero

**Appendix Table 6:** Difference-in-differences estimators from sensitivity analyses of the association between Medicaid expansions and outcomes, BRFSS, 2010-2020

|  | American Indian / Alaska Native | |  | White | |
| --- | --- | --- | --- | --- | --- |
| Outcome | B coefficient | (95% CI) |  | B coefficient | (95% CI) |
| **Sensitivity Analysis 1: Exclude early expanding states (DC, DE, MA, NY, VT)** | | | | | |
| Guideline-adherent cervical cancer screening | -0.01 | (-0.035 - 0.020) |  | 0.03 | (-0.000 - 0.062) |
| Ever had a pap test | -0.02 | (-0.075 - 0.029) |  | 0.00 | (-0.009 - 0.017) |
| Pap test in the last year | -0.02 | (-0.096 - 0.050) |  | 0.01 | (-0.021 - 0.046) |
| Pap test in the last 3 years | -0.00 | (-0.032 - 0.024) |  | 0.02 | (-0.005 - 0.047) |
| Pap test in the last 5 years | 0.01 | (-0.022 - 0.032) |  | **0.02** | **(0.004 - 0.039)** |
| Has a health plan | **0.06** | **(0.018 - 0.096)** |  | **0.12** | **(0.085 - 0.162)** |
| Avoided care due to cost | **-0.08** | **(-0.136 - -0.025)** |  | **-0.07** | **(-0.101 - -0.042)** |
| Had a checkup in the last year | 0.01 | (-0.055 - 0.083) |  | **0.04** | **(0.001 - 0.081)** |
|  |  |  |  |  |  |
| **Sensitivity Analysis 2: Exclude pregnant women** | | | | | |
| Guideline-adherent cervical cancer screening | -0.03 | (-0.071 - 0.016) |  | 0.03 | (-0.007 - 0.057) |
| Ever had a pap test | -0.03 | (-0.084 - 0.017) |  | 0.00 | (-0.015 - 0.010) |
| Pap test in the last year | -0.01 | (-0.093 - 0.064) |  | 0.00 | (-0.034 - 0.042) |
| Pap test in the last 3 years | -0.01 | (-0.043 - 0.025) |  | 0.02 | (-0.005 - 0.050) |
| Pap test in the last 5 years | -0.02 | (-0.060 - 0.012) |  | **0.02** | **(0.006 - 0.042)** |
| Has a health plan | 0.02 | (-0.040 - 0.069) |  | **0.09** | **(0.046 - 0.141)** |
| Avoided care due to cost | -0.08 | (-0.185 - 0.026) |  | **-0.05** | **(-0.090 - -0.019)** |
| Had a checkup in the last year | -0.01 | (-0.087 - 0.062) |  | 0.04 | (-0.005 - 0.085) |
|  |  |  |  |  |  |
| **Sensitivity Analysis 3: Exclude interviews in 365-day washout period** | | | | | |
| Guideline-adherent cervical cancer screening | -0.03 | (-0.066 - 0.007) |  | 0.03 | (-0.004 - 0.066) |
| Ever had a pap test | -0.03 | (-0.074 - 0.020) |  | 0.00 | (-0.011 - 0.018) |
| Pap test in the last year | -0.02 | (-0.106 - 0.065) |  | 0.01 | (-0.035 - 0.045) |
| Pap test in the last 3 years | -0.02 | (-0.048 - 0.018) |  | 0.02 | (-0.008 - 0.054) |
| Pap test in the last 5 years | 0.00 | (-0.029 - 0.030) |  | **0.03** | **(0.005 - 0.046)** |
| Has a health plan | **0.06** | **(0.018 - 0.107)** |  | **0.12** | **(0.070 - 0.159)** |
| Avoided care due to cost | -0.06 | (-0.126 - 0.015) |  | **-0.08** | **(-0.112 - -0.049)** |
| Had a checkup in the last year | 0.03 | (-0.031 - 0.085) |  | 0.03 | (-0.016 - 0.078) |
|  |  |  |  |  |  |
| **Sensitivity Analysis 4: Apply BRFSS survey weights** | | | | | |
| Guideline-adherent cervical cancer screening | -0.01 | (-0.090, 0.076) |  | 0.01 | (-0.023, 0.035) |
| Ever had a pap test | 0.01 | (-0.056, 0.074) |  | 0.00 | (-0.018, 0.023) |
| Pap test in the last year | -0.06 | (-0.156, 0.041) |  | 0.01 | (-0.023, 0.045) |
| Pap test in the last 3 years | 0.01 | (-0.070, 0.086) |  | 0.02 | (-0.005, 0.050) |
| Pap test in the last 5 years | -0.00 | (-0.074, 0.070) |  | 0.01 | (-0.009, 0.037) |
| Has a health plan | -0.01 | (-0.101, 0.074) |  | **0.11** | **(0.079, 0.141)** |
| Avoided care due to cost | -0.02 | (-0.113, 0.069) |  | **-0.06** | **(-0.092, -0.028)** |
| Had a checkup in the last year | **-0.11** | **(-0.206, -0.007)** |  | 0.03 | (-0.007, 0.061) |
|  |  |  |  |  |  |
| **Sensitivity Analysis 5: Exclude ages 18-24** | | | | | |
| Guideline-adherent cervical cancer screening | -0.02 | (-0.049 - 0.019) |  | 0.0 | (-0.003 - 0.051) |
| Ever had a pap test | -0.02 | (-0.071 - 0.029) |  | 0.0 | (-0.007 - 0.020) |
| Pap test in the last year | -0.02 | (-0.090 - 0.043) |  | 0.0 | (-0.022 - 0.045) |
| Pap test in the last 3 years | -0.01 | (-0.038 - 0.021) |  | 0.0 | (-0.006 - 0.048) |
| Pap test in the last 5 years | 0.00 | (-0.028 - 0.032) |  | **0.0** | **(0.004 - 0.041)** |
| Has a health plan | **0.04** | **(0.002 - 0.086)** |  | **0.1** | **(0.066 - 0.153)** |
| Avoided care due to cost | **-0.06** | **(-0.121 - -0.004)** |  | **-0.1** | **(-0.105 - -0.043)** |
| Had a checkup in the last year | 0.01 | (-0.060 - 0.081) |  | 0.0 | (-0.008 - 0.079) |
|  |  |  |  |  |  |
| **Sensitivity Analysis 6: Exclude non-reproductive ages (45-64)** | | | | | |
| Guideline-adherent cervical cancer screening | -0.03 | (-0.073, 0.010) |  | 0.02 | (-0.009, 0.055) |
| Ever had a pap test | -0.03 | (-0.084, 0.025) |  | 0.00 | (-0.016, 0.010) |
| Pap test in the last year | -0.03 | (-0.113, 0.044) |  | 0.00 | (-0.039, 0.039) |
| Pap test in the last 3 years | -0.02 | (-0.050, 0.015) |  | 0.02 | (-0.008, 0.049) |
| Pap test in the last 5 years | -0.02 | (-0.059, 0.010) |  | **0.02** | **(0.004, 0.040)** |
| Has a health plan | 0.01 | (-0.046, 0.072) |  | **0.09** | **(0.041, 0.135)** |
| Avoided care due to cost | -0.07 | (-0.174, 0.034) |  | **-0.05** | **(-0.086, -0.014)** |
| Had a checkup in the last year | -0.01 | (-0.078, 0.060) |  | 0.04 | (-0.010, 0.080) |
|  |  |  |  |  |  |
| **Sensitivity Analysis 6: Use preferred race variable as indication of AIAN race** | | | | | |
| Guideline-adherent cervical cancer screening | 0.00 | (-0.032, 0.038) |  | -- |  |
| Ever had a pap test | -0.01 | (-0.062, 0.034) |  | -- |  |
| Pap test in the last year | -0.02 | (-0.082, 0.052) |  | -- |  |
| Pap test in the last 3 years | 0.01 | (-0.032, 0.045) |  | -- |  |
| Pap test in the last 5 years | 0.02 | (-0.013, 0.049) |  | -- |  |
| Has a health plan | **0.05** | **(0.015, 0.093)** |  | -- |  |
| Avoided care due to cost | -0.05 | (-0.117, 0.017) |  | -- |  |
| Had a checkup in the last year | 0.01 | (-0.062, 0.085) |  | -- |  |

CI = confidence interval; BRFSS = Behavioral Risk Factor Surveillance System

Models are fully adjusted and include main effects for expansion status and a post expansion indicator (and their interaction) and are adjusted for state and year-specific unemployment rates (%) as well as individual level age group, income, education, dependent children, marital status, working status and included state and year fixed effects. Standard errors are clustered by state.

Bolded values indicate that the 95% confidence interval does not contain the null value of zero
